# Parent–Child Reminiscing about Past Pain as a Preparatory Technique in the Context of Children’s Pain: A Narrative Review and Call for Future Research

**DOI:** 10.3390/children7090130

**Published:** 2020-09-07

**Authors:** Maria Pavlova, Serena L. Orr, Melanie Noel

**Affiliations:** 1 Department of Psychology, University of Calgary, Calgary, AB T2N 1N4, Canada; mpavlova@ucalgary.ca; 2 Department of Pediatrics, Alberta Children’s Hospital, Calgary, AB T3B 6A8, Canada; Serena.Orr@albertahealthservices.ca; 3 Cumming School of Medicine, University of Calgary, Calgary, AB T2N 1N4, Canada; 4 Hotchkiss Brain Institute, Owerko Centre, Alberta Children’s Hospital Research Institute, Calgary, AB T3B 6A8, Canada

**Keywords:** preparation, parent–child reminiscing, memory for pain, pediatric pain, memory reframing

## Abstract

Pain permeates childhood and remains inadequately and/or inconsistently managed. Existing research and clinical practice guidelines have largely focused on factors influencing the immediate experience of pain. The need for and benefits of preparing children for future pain (e.g., painful procedures) has been well established. Despite being a robust predictor of future pain and distress, memories of past painful experiences remain overlooked in pediatric pain management. Just as autobiographical memories prepare us for the future, children’s memories for past pain can be harnessed to prepare children for future painful experiences. Children’s pain memories are malleable and can be reframed to be less distressing, thus reducing anticipatory distress and promoting self-efficacy. Parents are powerful agents of change in the context of pediatric pain and valuable historians of children’s past painful experiences. They can alter children’s pain memories to be less distressing simply by talking, or reminiscing, about past pain. This narrative review summarizes existing research on parent–child reminiscing in the context of acute and chronic pediatric pain and argues for incorporation of parent–child reminiscing elements into preparatory interventions for painful procedures.

## 1. Introduction

Pain is an inherent part of childhood. From birth, as a part of standard healthcare, children undergo numerous painful procedures (e.g., vaccine injections, venipunctures). The World Health Organization recommends that all children receive 10 or more routine immunizations by the age of 1 year [1]. Everyday pains (i.e., minor injuries) occur frequently and may be salient and distressing, despite rarely leading to serious harm [2]. Pediatric pain also occurs within the context of acute (e.g., bone fractures) and chronic (e.g., primary pain disorders, juvenile arthritis) conditions. Pain demands attention [3], disrupts normal functioning [4], and, if chronic, enmeshes with children’s social development [5], as well as physical [6] and mental health [7] trajectories.

There has been notable progress in pediatric pain management. Yet pediatric pain remains inadequately and/or inconsistently managed [8]. Existing research and clinical practice guidelines have largely focused on factors influencing the *immediate experience* of pain. Clinical interventions mirror this trend and guide the management of the child’s immediate pain experience. For example, the clinical practice guidelines on pain mitigation during vaccine injections recommends the “5P” approach (i.e., process, pharmacological, psychological, physical, and procedural intervention techniques), where most of the techniques focus on pain during vaccinations [9].

The importance of addressing future pain before it occurs (i.e., preparing children for a painful procedure) has been well established [10]. Preparatory interventions have been shown to result in reduced levels of pain and distress of children [10]. Children’s memory for *past* pain robustly predicts future distress and pain [11,12], such that children who remember more pain compared to their initial/earlier reports are more likely to experience greater distress and pain at future painful experiences. Despite the call [13,14] for harnessing the power of children’s memories for pain, pediatric pain management continues to overlook past painful experiences. Cognitive psychology research has posited an intriguing idea that episodic autobiographical memory (i.e., recall of specific past events) exists to help us imagine and prepare for the future [15]. Thus, capitalizing on the power of children’s autobiographical memories for past pain and incorporating these memories into preparatory interventions could open a novel avenue in preventative pain management.

Parents impact children’s pain experiences and can play an active role in reducing pain and distress before, during, and after painful experiences. Although current clinical practice guidelines [9] recommend that parents are present and actively involved in pediatric pain management (e.g., distracting their children), the role of parents as preparatory interventionists has not been extensively evaluated. Furthermore, the powerful potential of parent–child relationships has not been maximally utilized in the context of preparation for painful procedures. The few interventions that have included parents as the source of information provision demonstrated that parents are feasible and effective interventionists [16,17]. The existing interventions targeting children’s memories for past pain (i.e., memory reframing interventions) were delivered by adult researchers [18,19,20]. It has been argued that memory reframing should capitalize on parents’ key role in forming and altering children’s memories for pain [14]. One promising way to do that is to utilize parent–child conversations about past events (i.e., parent–child reminiscing). Developmental psychology research has demonstrated that parent–child reminiscing creates a robust socio-linguistic environment that shapes children’s autobiographical memories, as well as socio-emotional developmental outcomes (e.g., emotion understanding, emotion regulation skills) [21]. An emerging body of research has shown that parent–child reminiscing about past pain differs from reminiscing about other distressing events [22] and predicts how children remember their past pain [23]. The first randomized controlled trial of parent-delivered memory reframing intervention to alter children’s memories for past pain is underway [24].

This narrative review argues for incorporating parent–child reminiscing into preparatory interventions. Following a brief overview of conceptual models of preparation and information provision, as well as a review of the malleability of children’s memories for pain and parent–child reminiscing about past pain, the current paper elaborates on the benefits and risks of parent–child reminiscing as used in preparing children for painful procedures in the context of acute (e.g., vaccine injections, venipunctures) pain. This review also summarizes the available evidence of children’s memories for and parent–child reminiscing about pediatric chronic pain, as well as the applicability of reminiscing-enhanced preparation in the context of pediatric chronic pain. We conclude the review with a future research agenda to examine the efficacy of parent–child reminiscing in preparatory interventions.

## 2. Preparatory Interventions: An Overview of Theoretical Models

Information provision and preparation of children for painful procedures are guided by two key goals: (1) to provide children with information about the procedure; (2) to teach or remind children about effective coping skills [13]. Preparing children for painful experiences occurs in multiple formats (e.g., verbal explanations, instructional videos, behavioral modeling, hospital tours) and across multiple contexts (e.g., vaccinations, medical procedures, dental procedures, surgery). Provision of relevant information about, and encouraging children and parents to use coping skills during, painful procedures influence children’s distress levels *and* are easy to implement in healthcare settings. Preparatory interventions for painful procedures reduce children’s levels of pre-procedural distress as well as their level of experienced pain [10,25,26].

Central to understanding the merit of preparation and information provision is the concept of self-regulation, or one’s ability plan and adapt one’s behavior to reach an optimal outcome [27]. Self-regulation theory [28] was one of the first theoretical frameworks that explained the value of preparatory interventions in reducing illness-related distress. According to Johnson and Leventhal [28], how an individual copes with future distressing events is guided by two factors: (1) expectations about the future experience (e.g., a painful procedure) based on the individual’s experience of the same or a similar situation; (2) available information about the future experience. The novel information about the procedure helps the individual to form an accurate idea, or schema, about the future event, which reduces uncertainty about, and incongruence between, the expected and actual experiences. Johnson and Leventhal proposed two alternative explanations of how information about future events may contribute to coping behaviors. Specifically, Leventhal [29] proposed that information allows the individual to appraise the situation differently and, as a consequence, have higher levels of perceived control, resulting in less distress and adaptive coping. Johnson [30], on the other hand, hypothesized it was the comparison between information about a future event and past experiences that elicited more adaptive coping. For example, if a person experienced a similar event in the past, they would have more experience and examples of coping behaviors used in this context; if an event is novel, more general past coping strategies would be used. Empirical research provided mixed support for Johnson’s view [17,30]. Thus, self-regulation theory underscores the importance of past experiences in coping with future distress.

For young children, the development of self-regulation is non-linear and undergoes critical changes in early childhood (i.e., between the ages of 3 and 7 years) [31]. Young children’s regulation strategies are often reactive, require support and attention of their caregivers, and center around their most often negative emotions, such as anxiety or fear [32]. Self-regulation requires higher-order cognitive and behavioral abilities (e.g., abilities to predict future states, recall relevant past experiences, and compare predicted, immediate, and past experiences) [31]. For example, a young child may regulate anticipatory distress by crying, clinging to parents, or repeatedly asking questions about the procedure to get their parent’s attention and seek comfort/support. Therefore, preparatory interventions should consider and/or target young children’s emotional distress and facilitate optimal co-regulation strategies that involve children’s caregivers. In contrast, an older child whose regulation has changed to be more self-reliant may benefit from preparatory interventions that teach new coping techniques that the child can use without caregiver’s help (e.g., deep breathing, distraction) or highlight past successful instances of coping with pain.

Another key aspect to consider is the degree of predictability that contributes to the individual’s self-efficacy, or one’s belief that they are able to deal with the situation. With regards to *stressful* future events, Miller [33] highlighted the difference between controllability (i.e., being able to do something about the future event) and predictability (i.e., knowing something about the future event). When the levels of predictability are higher, distressing events are perceived as less stressful and individuals feel more self-efficacious, even if the event remains uncontrollable. For young children undergoing painful procedures, controllability is often limited (e.g., parents and medical staff oversee and control the procedures, children might have to get certain procedures done, such as vaccinations or stitches after an injury); predictability, on the other hand, may increase as a result of information provision about the procedure.

The role of past experiences in preparation for future events is complemented by schema theories. Past experiences are crystallized into schemata, or general frameworks, that organize and store memories [34]. Preparatory information activates appropriate schemata to produce expectations about future events and guide behaviors. When the event is occurring, schemata may direct attention toward certain aspects of the event. For example, if a child’s schema about hospitals is based on annual flu vaccinations, the child will likely expect an injection during any hospital visit; and should an injection occur, the child may preferentially attend to the needle and needle-related pain and distress. For novel experiences (e.g., undergoing surgery for the first time), no schemata exist, or the existing schema (e.g., “hospital”) may be too general or misleading. Preparatory interventions refine existing memories and cognitive frameworks to guide the experience. Importantly, young children’s memories and schemata are fragile, malleable, and susceptible to distortions [35]. If a health- or pain-related schema is distorted with distressing memories, it may be insufficient to simply provide specific details about an upcoming procedure. Children’s memories may first need to be altered to address the distortions and distress.

Jaaniste and colleagues combined the self-regulation and schema theories into the Information Provision Theory that details how preparatory information interacts with children’s past experiences to change the appraisal of, and reactions to, future medical procedures [36]. According to the model, provision of information triggers existing health- and pain-related schemata that, in turn, may be detailed and specific (if a child has had many similar procedures) or generic and lacking necessary details (e.g., pre-surgery preparation for a surgery-naïve child). Preparatory interventions aim to finesse the existing schemata to reduce anticipatory distress and promote self-regulation and optimal coping. Based on the refined schemata, children re-appraise the future procedure, adjust their expectations about potential pain and distress levels, and engage in appropriate coping skills. Once the procedure begins, children can compare their expected and actual levels of pain to further adjust their coping behaviors. Procedural outcomes (e.g., experienced pain and distress) further adjust the activated schemata and are consolidated into episodic memories of the procedure. Schemata and episodic memories modify each other.

In summary, past experiences and associated memories play a key role in preparing children for future painful procedures. Preparation relies on coping and emotion regulation skills that children have learned and practiced in the past. Information provision and preparation activate children’s health- and pain-related schemata; yet those schemata may be negatively altered by previous experiences and need to be changed to be less distressing. Thus, preparing children for future pain should necessarily consider past pain and memories for it. Yet, existing preparatory interventions have not targeted children’s memories for past experiences, nor have they harnessed the malleable nature of autobiographical memories. In the next sections, we review key factors that shape children’s memories for pain and elaborate on how parent–child reminiscing can be used to alter those memories and interact with preparatory information to reduce children’s pain-related distress.

## 3. Children’s Memories for Acute Pain

Poorly managed acute pain is long remembered and results in an array of detrimental sequelae. For instance, 3-year-old children remembered their visit to an emergency department following an injury up to 10 years later [37]. Children undergoing painful medical procedures without proper analgesia experienced higher levels of subsequent procedural pain intensity and did not respond adequately to subsequent analgesia [38]. As an evolutionary adaptation (by signaling threat and avoidance of harm), acute pain experiences—through a constellation of biological, psychological, and social factors—may set the stage for the onset of chronic (i.e., lasting for longer than 3 months) pain. Pediatric chronic pain affects 11–38% of youth and is a growing and costly (USD 19 billion/year) “modern public health disaster” ([39,40,41,42]). One of the proposed mechanisms underlying the transition from acute to chronic pain (i.e., persistent post-surgical pain that is often used to model the transition from acute to chronic pain) is memory for pain and, specifically, remembering pain in a distressing or negatively biased way. For example, in a prospective study of youths undergoing major surgeries, youths who developed negatively biased pain memories reported higher levels of pain five months post surgery [12].

Memory biases are defined as higher (i.e., negative memory biases) or lower (i.e., positive memory biases) levels of recalled pain as compared to initial/earlier reports. For young children, recall of acute experimental pain has been demonstrated to be a more robust predictor of future pain intensity as compared to the initial pain report [11]. Negative biases in pain memories are associated with future procedural anxiety, worse medical compliance, and the development of chronic post-surgical pain [12,43,44,45]. Memories for pain are amenable to change and are influenced by biopsychosocial factors. For example, psychological factors that impact children’s memories for pain include trait anxiety, catastrophic thinking about pain, pain anxiety, and anxiety sensitivity [45,46]. Biological factors that influence the development of pain memories have only been studied in adult samples and include, for instance, sensitization, or increased responsiveness, to painful stimuli that occurs on cellular and structural (i.e., spinal cord) levels and provides one of the neurobiological cornerstones of memory for pain [47]. Importantly, cognitive psychology researchers posit that the memory of past events is necessary to imagine the future [15]. For instance, thinking about the future and remembering the past activate the same neural circuits (i.e., medial temporal lobe, prefrontal cortex, and posterior cortices) [15]. Furthermore, predicting future pain, which inherently involves remembering past pain, may lay a neural foundation for the actual sensation of pain. For example, Koyama and colleagues demonstrated that predicting how much pain one would feel activated cortices (i.e., primary somatosensory cortex, insular cortex, and anterior cingulate cortex) associated with sensory pain experience [48].

The social factors influencing pain and memories for pain are a subject of an emerging, timely, and provocative discussion in the pain community. The social aspects of the pain experience have only recently been added as a note to the revised International Association for the Study of Pain definition of pain (i.e., “An unpleasant sensory and emotional experience associated with, or resembling that associated with, actual or potential tissue damage” and “Pain is always a personal experience that is influenced to varying degrees by biological, psychological, and social factors”) [49]. Although social modulation of pain behaviors is gaining increasing attention by basic and clinical researchers alike [50], investigation of the social factors influencing pain memories is lagging behind. Yet the social context, specifically parental influences, is of unequivocal importance for children’s and adolescents’ pain memory development. For example, parent psychological characteristics (e.g., higher levels of parent anxiety, parent catastrophic thinking about their child’s pain) were associated with more distressing children’s pain memories following surgery and were more predictive of children’s pain memories than children’s own anxiety [51,52]. Importantly, narrative exchanges between parents and children about past painful experiences—through reminiscing—are posited to be the most critical social context within which children’s acute and chronic pain memories are recalled, shared, and reconstructed [53].

## 4. Parent–Child Reminiscing

The broader field of developmental psychology has long demonstrated the pivotal role that parent–child reminiscing plays in children’s developmental trajectories. These parent–child narrative exchanges about past autobiographical pleasant and/or distressing events create a unique and influential milieu that allows children to share their recollection of past events and co-construct a meaningful and coherent account of the past together with their parents. Indeed, as Eric Kandel put it, “Recall of memory is a creative process. What the brain stores is thought to be only a core memory. Upon recall, this core memory is then elaborated upon, reconstructed, with subtractions, additions, elaborations, and distortions” [54]. Basic and clinical neurobiological research has shown that recalling a past experience makes the memory more vulnerable to distortions, with additional details incorporated or removed [55]. This vulnerability is starting to be harnessed in interventions. For example, using noradrenergic beta-blockers (e.g., propranolol) immediately following recall of a traumatic experience in adult patients with post-traumatic stress disorder disrupted memory reconsolidation and resulted in reduced fear symptoms [56]. Children, particularly young children, are highly suggestible, and their memories are particularly vulnerable to distortions [35,57]. Yes–no and leading questions, as well as reiteration of inaccurate details of a past event may result in false memories or merging of a child’s memory for a particular event with externally added details [35]. Children’s autobiographical memories are vulnerable to those distortions, especially when inaccurate information is—intentionally or unintentionally—relayed by children’s parents [58]. Thus, it is through narrative exchanges with parents that children’s memories, including those of past pain, are reconstructed, elaborated upon, and positively or negatively distorted.

Parent–child reminiscing shapes children’s autobiographical memory development. Young children of mothers who reminisced with them using open-ended questions containing novel emotion-rich details (i.e., elaborative reminiscing style) demonstrated better developed autobiographical memory skills at 3.5 years of age (i.e., recalled more unique details about a unique happy past event) [59,60]. Elaborative reminiscing, as opposed to topic-switching reminiscing (i.e., repeating the same information, asking closed-ended questions, talking about facts versus emotions), has also been linked to better-developed language skills [61], literacy skills [62], emotional understanding [63], emotion regulation skills [64], and theory of mind [65]. In sum, elaborative reminiscing is thought to be an adaptive way of talking and processing past events, as compared to the topic-switching reminiscing style that is conceptualized as non-adaptive.

A substantial amount of reminiscing research has focused on children’s memories of positive events (e.g., going on a vacation). Yet it is parent–child reminiscing about past distressing events (e.g., natural disasters, sadness, injuries) that is particularly powerful and beneficial [66]. As distressing events occur, parents and children may experience an array of negative emotions (e.g., fear, panic, frustration) and thoughts (e.g., catastrophizing, rumination). However, given the temporal remoteness from the distressing situation, reminiscing allows parents and children to reflect upon, process, and coherently re-construct past distressing experiences to arrive at a meaningful resolution in a safe environment. Parents may also use reminiscing to emphasize and rehearse adaptive coping strategies, thus expanding children’s coping repertoires. When talking about past distressing events (e.g., a visit to an emergency department following a minor medical trauma), parents tend to explain and elaborate (i.e., use open-ended detail-rich questions) more, as compared to discussing a positive past event [60]. Children of parents who used an elaborative, versus topic-switching, reminiscing style recalled their visit to an emergency department more accurately and completely (i.e., provided more unique units of information) [67]. A body of research examining the accuracy of children’s memory for stressful (often painful, e.g., a face laceration or bone fractures) events have demonstrated that children tend to remember details of those events accurately [61,67,68,69,70]. Yet it is children of elaborative reminiscers that remember the broader context of the event (i.e., hospital treatment that followed the injury) in more detail [67]. Given its unique and robust contributions to children’s autobiographical memory development, parent–child reminiscing about past painful experiences offers novel avenues in the study and advancement of pediatric pain management.

## 5. Parent–Child Reminiscing in the Context of Memories for Acute Pain

While implicit memories (i.e., unconscious or automatic memories) can be formed from birth, it is the explicit memory system (i.e., ability to consciously recall past events) that expands dramatically during the toddler and preschool years. Starting from the age of 3 years, children are rapidly developing the cognitive and verbal capacities that are needed to form, elicit, and discuss explicit memories of past pain. The first pain-related words (e.g., “Ouch”, “Ow”) appear in vocabularies of 17–18-month-old children; the pain vocabulary expands and develops during preschool years [71]. Preschool children are able to provide general and specific pain labels for vignettes describing an injury [72]. In another study, 3- to 6-year-old children were able to describe a picture of an acute pain injury and express how they would talk to their parents about a similar painful injury later [71]. Parents and young children frequently talk about pain (i.e., pain-related words were present in 29% out of 192 randomly sampled transcripts of everyday parent–child conversations) [71]. Yet, the majority of these conversations feature either present or future/imaginary pain, with only 2% of transcripts referring to past pain [71]. Similarly, the field of pediatric pain has extensively examined parent–child verbal exchanges in the immediate presence of pain [73], whereas research on parent–child conversations about past pain, while inevitably important for children’s understanding and experience of immediate or future pain, is scarce.

An emerging body of research has demonstrated the unique characteristics of parent–child reminiscing about past pain and its role in the development of children’s memories for pain. A recent observational study of preschool-aged children who underwent an outpatient surgery (i.e., tonsillectomy) demonstrated distinctive patterns of parent–child reminiscing about past painful versus sad events. When discussing the surgery versus a sad event, parents used reminiscing elements that characterized a topic-switching (i.e., less adaptive) reminiscing style. Specifically, parents used fewer explanations, open-ended questions containing new details, and words associated with negative emotions [74]. These reminiscing style elements have been associated with poorer developmental outcomes (i.e., worse emotion understanding, less prosocial behaviors, worse developed autobiographical memory skills) [60,75,76]. Another study with healthy 4-year-olds comparing parent–child reminiscing about past events involving past everyday pain versus sadness replicated these trends. When talking about past painful versus sad events, parents used fewer emotion-laden words, fewer explanations, but more coping- and pain-related words [22]. These findings suggest that children may be socialized to pain, compared to distressing emotions (e.g., sadness), differently and less adaptively via parent–child narrative exchanges. Furthermore, parent–child reminiscing about past painful events has been linked to children’s prosocial behaviors in response to another person’s pain. Parents who used open-ended questions containing new information, emotion-laden language, and discussed pain coping strategies when reminiscing about past everyday painful events had children who behaved more prosocially when witnessing a stranger in pain [22].

To date, there has been only one study investigating the role of parent–child reminiscing about past painful events and children’s pain memory biases [23]. Young children who recently underwent tonsillectomy talked about the surgery with their parents two weeks after the surgery (i.e., a time point when post-surgical pain usually subsides). One month after the surgery, children recalled how much pain and pain-related fear they experienced after the surgery. If parents used more positive emotion words (e.g., happy, laugh) and less pain-related words (e.g., hurt, owie) when reminiscing about the surgery, their young children developed more accurate and/or positively biased recall of their post-surgical pain [23].

The parent reminiscing style is malleable and can be changed using simple narrative interventions. For example, parents of young children were taught to reminisce using more open-ended questions and emotion-rich language [77]. Their children also demonstrated better developed autobiographical memory skills and understanding of emotions [77]. A recent review summarized the existing intervention studies aimed at changing parent–child reminiscing styles and demonstrated their effectiveness and long-lasting impact on children’s socio-emotional functioning [78]. Yet, despite the prominent role of parent–child narrative interactions in a conceptual model of children’s pain memory development [53], no studies to date have targeted parent–child reminiscing as a way to positively alter children’s memories for pain.

Just as parent–child reminiscing is malleable, so too are young children’s memories for pain. Given the critical importance of biased pain memories in children’s future pain trajectories (e.g., biased pain memories have been posited to be one of the factors that contributes to the transition from acute to chronic post-surgical pain) [12], there has been a call for harnessing the fragility of pain memories in pediatric pain management [14]. To date, there have been three randomized controlled trials [18,19,20] of memory reframing interventions to alter children’s memories for needle pain. Children received interventions following a needle procedure (i.e., lumbar puncture [20]; dental injection [19]; vaccine injection [18]). These brief interventions involved identifying positive aspects of the pain experience, adjusting negative biases in memory, and emphasizing children’s ability to cope with pain (e.g., commending their bravery, identifying instances where the child coped effectively).

Memory reframing can serve to reduce children’s anticipatory distress. Yet, only one of the memory reframing interventions addressed its influence on future pain-related distress. Chen and colleagues measured the effect of their memory reframing intervention on the levels of children’s anticipatory distress in the context of repeated lumbar punctures for cancer treatment [20]. Children in the intervention versus control group reported less anticipatory distress, as well as demonstrated reduced physiological anticipatory distress (as measured by heart rate) [20]. In an observational study, Marche and colleagues used retrieval-induced forgetting to investigate children’s ability to forget the distressing aspects of past painful experiences [79]. Focusing on and repeatedly rehearsing positive aspects of a past painful event that children identified led to them forgetting distressing details of the painful experience [79]. Importantly, children who had more difficulties forgetting negative aspects of the past painful events experienced more anticipatory anxiety prior to an experimental pain task (i.e., cold pressor) [79]. Overall, memory reframing principles (that can be readily incorporated into parent–child reminiscing) can be used to reduce children’s anticipatory distress and anxiety, which is the key objective of preparatory interventions.

To date, memory reframing interventions have all been conducted by adult researchers. Parents, who are powerful, yet underutilized, agents of change and valuable historians in the context of children’s memories, have not yet been involved in the delivery of memory reframing interventions [14]. The only parent-delivered reminiscing intervention is underway [24]. The intervention teaches parents of young children the key principles of pain memory reframing: (1) focusing on the positive aspects of a past painful experience; (2) decreasing any exaggerations in children’s recall of pain and/or pain-related fear; (3) emphasizing pain coping strategies and children’s pain-related self-efficacy [24]. The intervention elements directly relate to, and complement, preparatory interventions.

It is important to consider individual differences that influence children’s memories for pain. As posited by cognitive theories, anxiety and anxiety-related constructs play a pivotal role in development of memory biases, such that individuals with higher levels of anxiety preferentially attend and recall threatening information [80]. For example, children aged 8–12 years with higher state anxiety developed more negatively biased pain memories following an experimental pain task (i.e., cold pressor) [46]. Furthermore, children with higher trait anxiety and anxiety sensitivity (i.e., fear of bodily symptoms associated with anxiety, such as rapid heart rate) developed negatively biased memories for pain-related fear associated with the cold pressor task [46]. In a cohort of youths undergoing a major surgery, higher baseline and post-surgical (i.e., first 48 h after the surgery) levels of pain catastrophizing and anxiety sensitivity led to negatively biased memories for post-surgical pain [45]. Pain-related self-efficacy (i.e., one’s belief that they can cope with pain) is another factor that contributes to children’s memories for pain. In a study of retrieval-induced forgetting, where children rehearsed positive aspects of a past painful experience and, as a result, forgot negative aspects of that experience, children with lower levels of pain-related self-efficacy forgot fewer negative aspects of past pain and had higher levels of anticipatory distress prior to an experimental pain task [79]. Similarly, in a cohort of youths undergoing a major surgery, lower levels self-efficacy (i.e., higher levels of helplessness) predicted greater emotional distress during the acute post-surgical recovery (i.e., first 48 h post surgery), which, in turn, led to the development of negatively biased pain memories two–four months after the surgery [52]. The negative biases in pain memories predicted higher levels of post-surgical pain [12].

## 6. Risks and Benefits of Using Parent–Child Reminiscing in Preparatory Interventions for Procedural Pain

As outlined in the sections above, preparing children for future painful events necessarily relies on their memories for past pain that are malleable, susceptible to distortions, and can be altered to be less distressing. One way of altering children’s memories for pain is by reminiscing with them about past painful events and highlighting positive aspects of the experience, helping them re-appraise any distressing and/or negatively biased memories, and reminding them about coping strategies they used to increase self-efficacy. Parent–child reminiscing about past pain can be readily incorporated in preparatory interventions and enhance specific aspects of the interventions.

First, an elaborative parent reminiscing style (i.e., using open-ended questions and providing new and/or forgotten details about the past painful event) may refine and add to children’s existing pain-related schemata. Children who are distressed during painful procedures remember fewer details about the procedure [81]. Furthermore, memories for distressing events are not a mere sum or an average of the experienced distress; instead, those memories usually center around the most salient details of the event (i.e., the highest level, or peak, of distress as well as distress during the last moments of the event, also known as the peak–end phenomenon [82]) whereas minor details may be forgotten [83]. For instance, a child’s memory for a painful vaccination may focus on the needle and pain, whereas the coping skills that the child and/or parent used during the experience (e.g., using a numbing cream) may be overshadowed by the pain-related distress and therefore lost. The parent would be in a uniquely advantageous position to reconstruct the memory with the child prioritizing positive aspects and self-efficacious coping strategies. Reframing the last and peak distressing moments of the vaccination to be less negatively biased may alter the overall memory for the vaccination to be more positive and contribute to children’s future behavioral outcomes. In a seminal study, the last moments of an invasive and painful medical procedure (i.e., colonoscopy) were made less painful, rendering more accurate/less negatively biased memories and greater future medical adherence (i.e., repeat colonoscopy) [84]. Reminiscing with the parent after the event enriches the child’s memory for the event and adds coping skills to the child’s behavioral repertoire. As a result, children’s self-efficacy and their perceived control and predictability in relation to future painful procedures increase. Reminiscing once again before the next vaccination would activate the enhanced child’s schema and remind the child about the learned coping skills. Furthermore, parents can reminisce with their children multiple times after the procedure. In the context of repeated painful procedures, parents can incorporate details of those procedures into an ever-evolving narrative with more and more evidence for children’s coping with, and bravery in the face of, pain.

Second, young children may shift their self-regulation from higher-order cognitive to emotion regulation strategies. A stranger (e.g., a nurse) providing information to a young patient may not pick up on the child’s distress and/or the need for emotion regulation. The elaborative reminiscing style has been linked to children’s better-developed emotion regulation skills [64]. Emotion-rich language of elaborative reminiscing and validation of children’s emotions may support and enhance children’s emotion self-regulation prior to a painful procedure and decrease the levels of distress, ultimately leading to less pain and pain-related fear during the procedure.

Third, parent–child reminiscing promotes parents’ involvement in pediatric pain management. Parents are highly capable and efficacious preparatory interventionists [17]. The principles of optimal reminiscing about past pain can and should be used by other adults involved in the provision of painful procedures (e.g., nurses) to prepare children for future painful procedures. In this context, parents could be valuable historians and provide information about their past painful procedures (e.g., positive aspects, coping and self-efficacy promoting strategies used) that could be used for memory reframing with children. Additionally, adaptive reminiscing about past pain may reduce the distress of parents, which is a crucial factor in pediatric pain experience and management. When reminiscing about a past painful event in a way that highlights its positive aspects and alters any distressing memories, parents themselves engage in retrieval-induced forgetting, where the negative or scary aspects of the past are forgotten by the virtue of rehearsing the positive aspects [79]. Reminiscing about available coping skills may increase parents’ pain-specific self-efficacy as well as encourage their advocacy for better pain management for their children.

Parent–child reminiscing should be implemented in preparatory interventions with a degree of caution. First, when talking about past pain, parents tend to use the elements of the topic-switching reminiscing style (e.g., repeating information, not using emotion-laden words) [22]. Repeatedly reminding children about the most salient and, most likely, distressing element of the past painful experience (e.g., a needle) may prompt more distress and distract children from the procedural information (i.e., what is going to happen during the procedure).

Another factor to consider is a potential incongruence between the degree of pain that the child expects, based on reminiscing, and their actual experiences during a procedure. A key part of preparatory interventions is the provision of *accurate* sensory information (i.e., how much pain a child would feel during the procedure). At times, expected levels of pain may be moderate to severe, yet a child may have an inaccurate estimation of the expected pain, particularly in cases of novel procedures [10]. In a trial of a preparatory intervention for young children undergoing ear piercings for the first time, children in the intervention, compared to control, group adjusted their pain expectations measured before and after the intervention to a greater degree (i.e., children who expected severe levels of pain *and* children who expected low levels of pain adjusted their expected pain to moderate) [17]. Furthermore, children who underestimated their pain subsequently experienced more pain during the procedure [17]. It is also important to consider that procedures with low levels of expected pain may be experienced as moderately or severely painful by some children. Inadvertently, when reminiscing about past pain, parents may diminish the child’s levels of past pain, thus creating a larger incongruence for the child. However, optimal and ethical parent–child reminiscing will not deny or lessen children’s experience of past pain. Opposite to some of the previous memory reframing interventions where researchers told children they did not cry regardless of whether or not it was true [18], parents are encouraged to use the factual information (i.e., what the parent witnessed and what the child experienced) and concentrate on adjusting any negatively biased and distressing pain memories to be more accurate.

Finally, schemata theories posit that using pain-related words may activate children’s pain-related schemata, such that children may be expecting, and preferentially attending to, pain and pain-related stimuli in the environment. A recent study demonstrated that frequent use of pain-related words when reminiscing about past post-surgical pain resulted in children remembering their pain in a negatively biased way [23]. Thus, when reminiscing about past painful experience before a painful procedure, parents should avoid using pain-related words and instead focus on discussing factual/accurate information, coping strategies used, and positive aspects of the past painful experience.

## 7. Future Research and Clinical Directions

This area of inquiry is ripe for future empirical investigation and clinical innovation. The research agenda is to examine several aspects of incorporating parent–child reminiscing into preparatory interventions. First, pilot trials need to assess the feasibility and acceptability of including parent–child reminiscing into preparation for painful procedures in healthcare and community (e.g., school-based vaccinations) settings. Comparative efficacy trials should investigate whether reminiscing-enhanced preparation is superior to preparation alone. Research should also examine the efficacy and comparative effectiveness of differently timed and combined interventions. For example, it could be that reminiscing after a painful procedure combined with reminiscing before the next painful procedure is more effective than reminiscing just before or just after the procedure alone. We would hypothesize that a combination of repeated reminiscing and provision of preparatory information would result in the greatest decreases in procedural pain.

Second, preparatory interventions often involve various media (e.g., videos, pamphlets, demonstrations using puppets). In addition to reminiscing principles, existing memory reframing interventions use visual tokens to remind children about their coping skills and self-efficacy (e.g., a colorful card to remind children to think about their coping skills [20]; a picture of the child during the dental procedure [19]). Future research should examine different media and their combinations for reminiscing and preparatory interventions.

Parents are effective preparatory interventionists [17] and are children’s main reminiscing partners [21]. They are likely to recall key information about children’s past painful procedures. This parent-provided information may be used by clinicians (e.g., nurses, physicians) to engage in reminiscing with children as a part of preparation for the upcoming painful procedure. For example, a nurse can have a brief conversation with a parent about their child’s most recent painful procedure and obtain relevant information to use while reframing. The nurse could then reiterate the most helpful coping strategies that the child used in the past (e.g., deep breathing) and enhance the child’s belief in their ability to cope with pain by reminding them how well they coped in the past and therefore how well they will cope with the pain today. Immediately following the procedure, clinicians can also make suggestive statements, based on the child’s coping and bravery just displayed, about how well the child will cope with similar procedures in the future. Parents, who typically present with children during painful procedures, can actively participate in this memory reframing with clinicians. The feasibility, acceptability (as perceived by both parents and healthcare providers), and efficacy of such parent-informed clinician-delivered preparatory intervention needs to be examined.

It is important to examine parent role (i.e., mothers vs. fathers) and child sex differences as potential moderators in reminiscing-enhanced preparation. Information provision trials have not reported any differences in response to the intervention as a function of child sex. There are potential differences between fathers and mothers regarding information provision. Males focus on and engage in information-based communication, whereas females focus on providing emotional support [85,86]. Furthermore, women, compared to men, reported higher levels of self-efficacy in providing emotional support messages [85]. Compared to mothers, fathers may find it more challenging to talk about emotional aspects of pain when preparing a child for a painful procedure. Sex and parent role differences exist in parent–child reminiscing. For example, when reminiscing about a recent surgery, fathers used explanations (i.e., an elaborative reminiscing style element) more frequently as compared to mothers [23]. When discussing a past painful event, parents used more questions containing novel information and talked more about coping strategies with boys versus girls [22]. Drawing causal relationships, enriching memories with new and/or forgotten details, and discussing coping skills are likely to better prepare children for future pain. Therefore, reminiscing and preparatory interventions may need to be tailored to mothers and girls to ensure that the key beneficial elements are included.

Future research should examine other potential moderators of reminiscing-enhanced preparatory interventions. Individual differences that are influential in the formation of pain memories and cognitive schemata about pain are likely to impact the efficacy of reminiscing-enhanced interventions. Children who tend to develop negatively biased memories for pain may benefit more from the combination of memory reframing reminiscing and information provision, as compared to children with accurate or positively biased pain memories. Children with high levels of anxiety-related constructs (e.g., anxiety sensitivity, pain catastrophizing) may develop distressing pain memories that are resistant to change as a result of one instance of reminiscing, and a preparatory intervention may trigger those distressing pain memories, leading to anticipatory distress. These children may benefit from repeated reminiscing interventions in combination with reminiscing-enhanced preparation to alter anxiety-driven distressing memories. For children with low levels of self-efficacy, reminiscing about past pain and preparation for future procedures will likely need to repeatedly emphasize and enhance their pain-related self-efficacy (e.g., providing concrete examples and reminders of how well the child coped with past pain).

A prominent area of future research concerns the use of parent–child reminiscing in the context of pediatric chronic pain and preparation of youth living with chronic pain for painful exercises and procedures. The role, origin, and prognostic value of pain memories in the context of primary pain disorders (e.g., recurrent headaches and/or migraines, complex syndrome regional pain) is unclear. The existing studies of memory in the context of pediatric chronic pain utilized methods from acute pain memory research (i.e., repeated use of single item pain intensity scales) and focused on the accuracy of and biases in pain recall [87,88,89]). This is clearly a methodological challenge in a context where pain is ongoing, often for years. Given mixed findings with regards to memory biases, and no reported links to subsequent outcomes, it seems that the study of memory in pediatric chronic pain has reached an impasse. This is surprising given the critical role of memory in conceptual models of pediatric chronic pain [90]. We argue that a different method of investigating recall of past pain in pediatric primary pain conditions is needed to overcome the unidimensional nature of extant research. Parent–child reminiscing may offer a unique framework to examine how memory for chronic pain is recalled, constructed, and reconstructed, which, in turn, could prepare children for future pain and lead to better pain outcomes. Furthermore, it is important to consider that parent–child reminiscing essentially provides a snapshot of parent–child verbal interactions that are thought to play a powerful role in children’s pain outcomes [91]. Reminiscing provides an idiographic representation of memory for salient past pain experiences, and how these memories are communicated within the parent–child relationship.

Reminiscing also has the potential to improve care through novel intervention strategies. For example, reminiscing may be used to encourage participation in nonpharmacologic treatments for chronic pain. Physiotherapy and exercise therapy are among the most effective nonpharmacological strategies for managing pediatric chronic pain and are a key component of multidisciplinary rehabilitation programs [92]. However, physical exercises are often aversive due to increased pain levels, which can exacerbate a youth’s fear and avoidance of exercise and physical therapy. Exercise and physical therapy are commonly delivered alongside cognitive-behavioral and/or acceptance- and commitment-based interventions [92]. Therefore, elements of reminiscing that target a youth’s pain specific self-efficacy and past coping strategies (e.g., highlighting how well the youth did last time, emphasizing how much better the youth felt after the previous physical therapy session) can be readily incorporated into preparing and motivating the youth for physical and/or exercise therapy. Similarly, parent–child reminiscing may be helpful for children with chronic migraines and/or headaches, who often have to undergo painful medical procedures (e.g., nerve blocks [93], intravenous injections in emergency settings [94]) to manage their pain. An enhanced ability and readiness to engage in potentially painful activities (e.g., exercises) or painful medical procedures (e.g., nerve block) may then generalize to other areas of functioning that is often associated with increased pain (e.g., return to school) and boost re-engagement in activities of daily living (e.g., playing sports, socializing).

## 8. Conclusions

Preparation and information provision in the context of pediatric pain is a burgeoning area of inquiry that has contributed to, and holds enormous potential to enhance, pediatric pain management. To date, preparation of children for painful procedures has focused on the management of initial expectations, refinement of and adjusting existing health- and pain-related schemata, and modifying appraisals of future pain procedures. The existing preparatory interventions have not yet utilized or targeted children’s past experiences and past learning, which forms the basis for children’s pain-specific expectations and schemas. Only a few preparation techniques involve parents, who are underutilized agents and overlooked stakeholders in the pediatric pain context. Parent–child reminiscing about past painful experiences represents a unique ready-to-implement preparatory intervention technique. Parent–child reminiscing about past pain has unharnessed potential to remind children about positive aspects of their past painful experiences, highlight the most effective coping skills that children and/or parents used in the past, and reframe any negatively biased or distressing memories for past pain that children may have. Needle procedures are a prime context to apply the principles of parent–child reminiscing as a preparatory intervention. Nearly every child has experienced needle-related pain; therefore, parents can reminisce with their children about past needle procedures at any point and reframe any negatively biased memories and, in doing so, parents can prepare their children for future needle procedures. Novel painful procedures (e.g., catheterization, surgery) may require more generalized reminiscing about past pain with a focus on coping strategies and children’s pain-specific self-efficacy. By fostering self-efficacy and accurate and positive memories, this simple language-based intervention holds potential for not only improving future pain experiences but also reducing avoidance of healthcare and vaccine hesitancy. In the context of pediatric chronic pain, reminiscing may increase a youth’s willingness and readiness to engage in therapeutic yet painful interventions (e.g., physical/exercise therapy) and activities of daily living. Thus, parent–child reminiscing creates a safe and empowering context for children to face and deal with future pain and pain-related distress and, hopefully, for them to experience pain and distress to a lesser degree.
